# Paediatric Strongyloidiasis in Central Australia

**DOI:** 10.3390/tropicalmed3020064

**Published:** 2018-06-13

**Authors:** Angela Wilson, Deborah Fearon

**Affiliations:** 1 BBioMedSci MBBS Hons, Paediatric Senior Registrar, Department of Paediatrics, Alice Springs Hospital, P.O. Box 2234, Alice Springs NT 0871, Australia; 2 FRACP, Head of Department, Department of Paediatrics, Alice Springs Hospital, P.O. Box 2234, Alice Springs NT 0871, Australia; Deborah.fearon@nt.gov.au

**Keywords:** strongyloidiasis, *Strongyloides stercoralis*, Indigenous, child health, Aboriginal and Torres Strait Islander, epidemiology, Central Australia

## Abstract

Few published studies are available describing the prevalence of paediatric strongyloidiasis in endemic areas within Australia. This literature review and exploratory clinical audit presents the first seroprevalence data for paediatric patients in Central Australia. A total of 16.1% (30/186) of paediatric inpatients tested for *Strongyloides stercoralis* in 2016 were seropositive (95% CI: 11.5% to 22.1%). Eosinophilia of unknown aetiology was the most common indication for testing (91.9%). Seropositive patients were significantly more likely to reside in communities outside of Alice Springs (*p* = 0.02). Seropositive patients were noted to have higher mean eosinophil counts with a mean difference of 0.86 × 10^9^/L (95% CI: 0.56 to 1.16, *p* < 0.0001), although the limited utility of eosinophilia as a surrogate marker of strongyloidiasis has been described previously. All seropositive patients were Indigenous. There was no significant difference in ages between groups. There was a male predominance in the seropositive group, although this was not significant (*p* = 0.12). Twelve patients had known human T-lymphotropic virus 1 (HTLV-1) status and all were seronegative. Further research describing the epidemiology of strongyloidiasis in Central Australia is required.

## 1. Introduction

The soil-transmitted helminth *Strongyloides stercoralis* has been described as one of the most neglected of the neglected tropical diseases [1]. Globally, strongyloidiasis is estimated to affect 30 to 370 million people, although data are limited [2,3]. *S. stercoralis* can cause decades-long infection in human hosts [4]. Infection may be clinically silent or cause a range of respiratory, skin, and gastrointestinal symptoms, or fulminant hyperinfection, typically in the setting of immune compromise [5,6,7].

The *Strongyloides* genus includes over fifty species capable of establishing parasitic infections in a range of animal hosts, and two species are known to infect humans [8]. *Strongyloides fuelleborni* is present in Papua New Guinea and Africa, while *S. stercoralis* is endemic throughout southern Europe, Africa, Asia, the Americas, and the northern two-thirds of Australia [2,9].

Some remote Australian Indigenous communities have *S. stercoralis* seroprevalences approaching 60%, putting them amongst the highest in the world [2,10,11]. Within these communities, Indigenous children have a higher documented prevalence of strongyloidiasis than any other age group [9,12,13,14,15,16].

*S. stercoralis* disproportionately affects resource-poor populations [17]. Remote Indigenous communities face an inequitable burden of poor health, socioeconomic disadvantage, and barriers to environmental control that impair disease control at a population level [8,18].

Human T-cell lymphotropic virus type 1 (HTLV-1) is an oncogenic virus that infects CD4+ T cells and interferes with Th2 immune responses [19]. HTLV-1 is endemic in Central Australia, and co-infection with *S. stercoralis* is associated with severe strongyloidiasis, *Strongyloides* treatment failure, and increased likelihood of developing T cell lymphoma [5,7,20]. HTLV-1 prevalence in Central Australia is estimated to be approximately from 7.2% to 13.9% among Indigenous adults [20].

**Figure 1:**
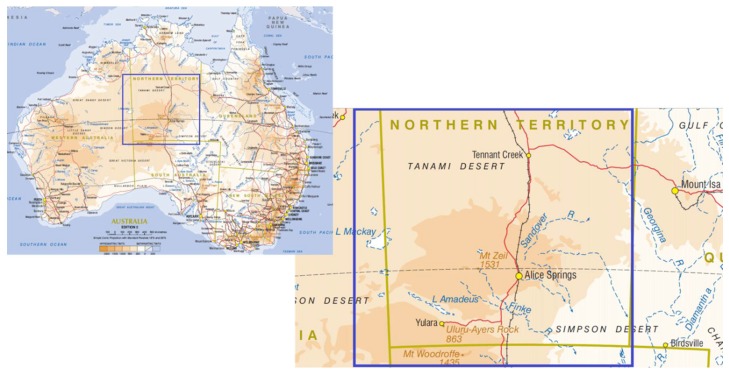
Approximate catchment area of Alice Springs Hospital [21,22].

Alice Springs Hospital services an extremely remote area of Central Australia that includes the southern half of the Northern Territory and adjacent parts of Western Australia and South Australia. It has a catchment area of approximately 900,000 square km with a population of 48,000 people, of whom 44% are Indigenous Australians (see Figure 1) [21].

Over the last three years, the paediatric department has increasingly tested patients with unexplained eosinophilia or other growth, respiratory, or abdominal symptoms for strongyloidiasis, and is in the process of formalising a policy to improve the recognition and management of this condition. HTLV-1 serology is performed on patients with clinical suspicion of immune compromise, particularly in children with chronic suppurative lung disease.

This paper will review of the literature relevant to *S. stercoralis* epidemiology in endemic areas of Australia, and present the results of an audit of *S. stercoralis* testing of paediatric inpatients at Alice Springs Hospital.

## 2. Review of Endemic Strongyloidiasis Epidemiology in Australia

*S. stercoralis* has been recognised as a pathogen in Australia for almost a century [23]. Studies examining the prevalence of strongyloidiasis in Australia can be divided into those undertaken in endemic areas and those describing prevalence in groups that have likely acquired it overseas (including migrants and refugee groups and returned military service personnel) [2]. This paper will focus on strongyloidiasis epidemiology in endemic areas within Australia.

The life cycle of *S. stercoralis* is complex and directly relevant to estimates of prevalence [24]. Male and female adults are capable of a single generation of free-living sexual reproduction outside of hosts, and non-infectious rhabditiform larvae moult into parasitic filariform larvae capable of surviving for up to two to three weeks in the environment under optimal conditions [25].

Filariform larvae penetrate host skin and migrate through the lymphatic or venous system to the lungs. They ascend the respiratory tree, are swallowed and migrate to the intestine. Parthenogenic female adults mature and invade the wall of the duodenum and jejunum where they lay up to fifty eggs per day [24]. Eggs hatch into rhabditiform larvae that migrate back into the intestinal lumen. Larvae may pass into the stool or mature into filariform larvae within the intestine and penetrate back into the host, establishing an auto-infective cycle [24].

A review of existing original research relating to the epidemiology of strongyloidiasis in endemic areas of Australia is presented in Table 1. This table is adapted from [11,18] with additional papers identified from Medline search and reference lists. Articles were located using Medical Subject Headings (MeSH) and text-word terms ‘Strongyloides’ or ‘Strongyloidiasis’ and ‘Australia’. Papers presenting epidemiological data from *S. stercoralis* endemic areas within Australia were included. Case reports and papers presenting data from other populations were excluded.

Estimates of strongyloidiasis prevalence within endemic areas in Australia vary widely depending on diagnostic method, population surveyed, and season. Community-based studies using faecal larval detection report prevalence rates from <1% to 41%, with substantial increases during the wet season in some locations [10,11,15,26,27]. Agar plate culture for a single stool sample is reported to be less than 60% sensitive [28]. Yield improves with multiple stool examinations and specialised microbiological techniques such as Baermann concentration [28], although this is not available at our health service.

Serology is more sensitive than stool detection of *S. stercoralis* larvae [28]. The sensitivities of various serological assays range from 75.4% to 93.9%, and specificities from 92.2% to 100% [29]. Flannery and White [30] reported the highest seropositivity rate in Australia of 59.6% of individuals tested in one small Northern Territory community. In Central Australia, Einsedel and colleagues reported a seroprevalence of 23.9% among 1126 hospitalised Indigenous adults [20]. No studies examining the seroprevalence of *S. stercoralis* in children in Central Australia were identified.

Children are over-represented in population estimates of strongyloidiasis. A Territory-wide study examining faecal larval detection between 2002 and 2012 found that children under five represented 42.2% of diagnoses, with rates of 3–6% of stool samples examined compared to 1.7% of samples overall [9]. A study of patients diagnosed with strongyloidiasis by faecal microscopy at Royal Darwin Hospital also identified that patients under five years of age were disproportionately represented, with 54% of cases falling in this age group [13].

Growth faltering remains a serious problem in the Northern Territory, affecting about 1 in 7 children under 5 years old in remote communities [31]. Associations between strongyloidiasis and malnutrition are well established but debate remains as to whether strongyloidiasis alone can cause growth faltering or represents an opportunistic infection in a compromised host [1]. The criteria for malnutrition were met by 80% of children diagnosed with strongyloidiasis in one study [13]. In another, Indigenous children with malnutrition were 6.5 times (95% confidence interval [CI]: 1.6 to 26.7) more likely to have *S. stercoralis* than a control group of well-nourished children [14].

Eosinophilia may be the only feature of strongyloidiasis in otherwise asymptomatic hosts, but remains an unreliable marker of strongyloidiasis. Mayer-Cloverdale and colleagues [9] found that just 40.8% of all patients with detectable *S. stercoralis* larvae in their stool had eosinophil counts of 0.5 × 10^9^ cells/L or greater. Eosinophilia was more common in patients under five and was present in 65.5% of positive cases (*p* < 0.0001) [9].

## 3. Clinical Audit Methods

Retrospective admission data from Alice Springs Hospital for the 2016 calendar year were obtained. The records of 2071 patients under the age of 16 years old admitted to the paediatric ward were reviewed as part of a departmental audit. Of these, 186 patients who had been tested for *S. stercoralis* were identified. Nonidentifiable coded data relating to patient demographics, clinical presentation, indication for testing, haemoglobin, mean corpuscular volume, eosinophil count, *Strongyloides* serology results, HTLV-1 status (if known), and faecal examination results were collated.

Symptoms at presentation were noted for each patient, with specific reference to growth faltering and gastrointestinal, respiratory, dermatological, and blood stream infections that might be attributable to strongyloidiasis. Growth faltering was defined as weight for age below the 3rd centile, standard weight for height less than two standard deviations below the mean, or crossing of two or more centile lines. Gastrointestinal symptoms included abdominal pain, altered bowel habit, vomiting, and anorexia. Respiratory symptoms included cough, dyspnoea, tachypnoea, chest pain, and pharyngitis. Dermatological manifestations were limited to urticarial rash or larva currens. Pruritus was not included due to the endemic nature of scabies and head lice in this population.

*S. stercoralis* serology was performed by Western Diagnostic Pathology, using an IgG enzyme-linked immunosorbent assay (ELISA) produced commercially by DRG Instruments. This assay detects IgG directed against the soluble fraction of filariform *S. stercoralis* larvae. The sensitivity of this assay is reported to be 91.2% with a specificity of 93.3% [37]. An optical density of 0.2 or greater is considered positive. In patients from nonendemic areas, a result of 0.2 to 0.4 is considered equivocal.

Statistical analysis was conducted using GraphPad software. Continuous data sets were analysed using unpaired *t*-tests. Confidence intervals for categorical data were calculated using the modified Wald method, and *p* values were determined using Chi-square calculations.

This study was conducted in accordance with the Declaration of Helsinki. No identifiable patient data was collected or retained by the investigators.

## 4. Results

Eosinophilia of unknown aetiology was the indication for testing in 91.9% (171/186) of patients, and seven were tested because of previous eosinophilia. Of the remaining patients, one patient had growth concerns, one was commenced on immunosuppressant medications, and six had gastrointestinal or respiratory presentations suggestive of strongyloidiasis.

**Figure 2:**
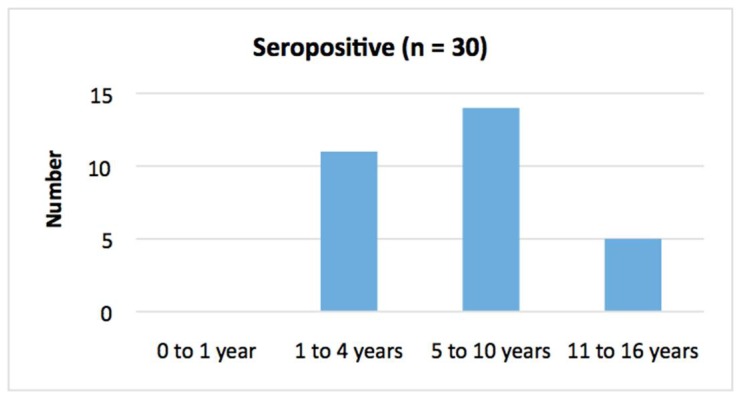
Age distribution in *S. stercoralis* seropositive group.

**Figure 3:**
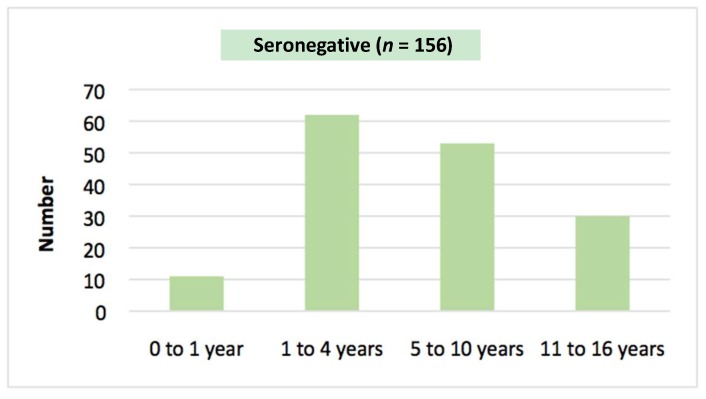
Age distribution in *S. stercoralis* seronegative group.

Overall, 16.1% (30/186) of patients tested were seropositive for *S. stercoralis* (95% CI: 11.5% to 22.1%). There was no significant age difference between seropositive and seronegative groups (*p* = 0.55) (Table 2, Figure 2 and Figure 3). A male predominance in the seropositive group was observed although the difference was not significant (*p* = 0.12). Seropositive patients were significantly more likely to reside in communities outside of Alice Springs (*p* = 0.02).

**Figure 4:**
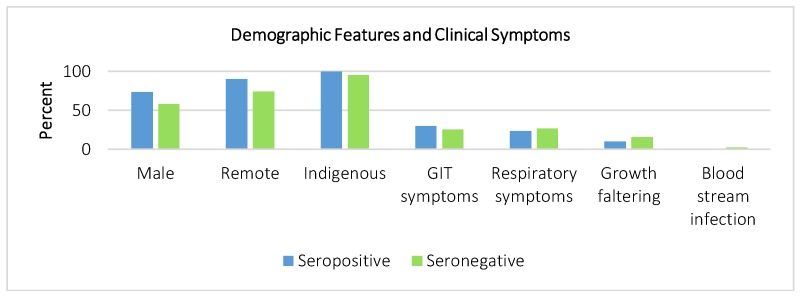
Clinical features during admission. GIT: Gastrointestinal tract.

The data did not support any significant differences in clinical presentation, haemoglobin, or mean corpuscular volume (Figure 4). Four seronegative patients had bloodstream infections, including one patient with cryptococcal disease and three patients with *Staphylococcus aureus* bacteraemia. No patients in either group presented with urticaria or larva currens. No cases of hyperinfection were identified, and none of the 12 patients who had HTLV-1 testing were seropositive.

Within the group of patients tested because of eosinophilia, seropositive patients were noted to have a significantly higher mean eosinophil count with a mean difference of 0.86 × 10^9^/L (95% CI: 0.56 to 1.16, *p* < 0.0001). Of the 55 patients that had a stool sample sent, none had *S. stercoralis* larvae detected (Table 3). There was no significant difference in the rate of other stool pathogens identified between groups (*p* = 0.09).

**Figure 5:**
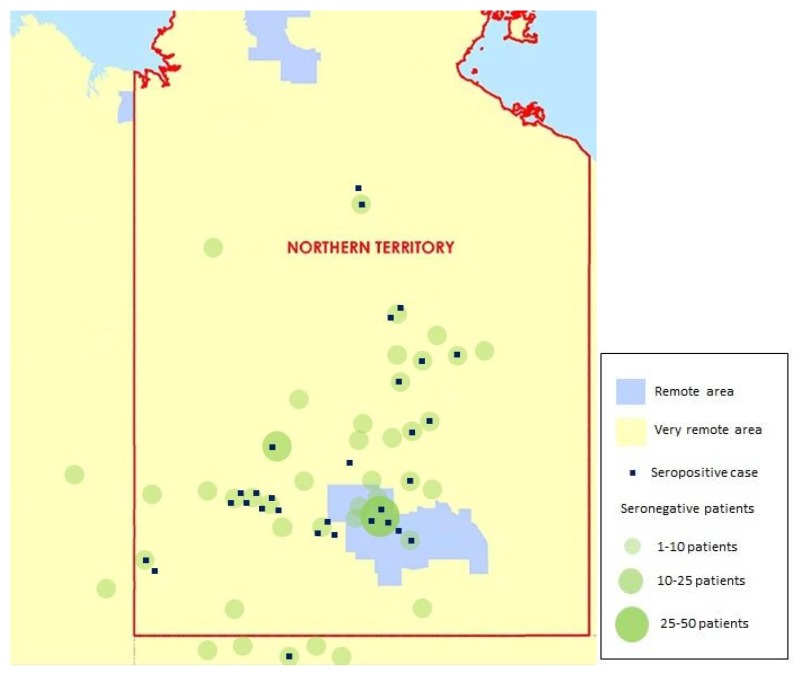
Geographical distribution of seropositive and seronegative cases in Central Australia.

The geographical distribution of seropositive and seronegative patients is shown in Figure 5.

## 5. Discussion

This exploratory audit highlights many of the universal challenges of understanding and managing *S. stercoralis*. Robust epidemiological data are lacking, clinical features and surrogate markers for infection are poorly sensitive and specific, and microbiological diagnosis is difficult. Although there was a significant difference in mean eosinophil counts, wide ranges and substantial overlap between data sets highlight the limitations of eosinophilia as a clinically useful indicator of possible strongyloidiasis. Further investigation is required to better understand the burden and epidemiology of strongyloidiasis in children in Central Australia.

This audit is limited by small patient numbers, retrospective data collection, and selective population sampling. No reliable conclusions regarding the prevalence of strongyloidiasis among the general paediatric inpatient population or paediatric population in Central Australia can be drawn from this audit. The geographical distribution of cases cannot be used to infer community prevalence but may suggest a clustering of cases in western and northern communities. This is also likely to reflect in part the relative distribution of the remote populations surrounding Alice Springs.

The predominance of remote diagnoses is likely to reflect the ability of *S. stercoralis* to thrive in infrastructure-poor areas, and strongyloidiasis remains a disease predominantly of the poorly resourced in Central Australia [17]. The social determinants of health are starkly relevant in this context, and Einsiedel and Fernandez summarise some of the challenges that remote Indigenous communities face in controlling strongyloidiasis at a population level [5]:

Ultimately, strongyloidiasis is a disease of poverty that reflects the appalling socioeconomic situation of Indigenous Australia. In some communities, a median number of 17 persons live in each house, and nearly 50% of dwellings do not have functioning facilities to remove faeces. The endemicity of both S. stercoralis and HTLV-1... renders public education and improvements to housing imperative.

Socioeconomic disadvantage is associated with higher rates of morbidity and mortality from strongyloidiasis, particularly where this leads to overcrowding, breakdown in sanitation systems, and environmental disease reservoirs from soil contamination [17]. Addressing water, sewerage, and garbage management systems remains fundamental to breaking the cycle of infection and reinfection [8].

**Figure 6:**
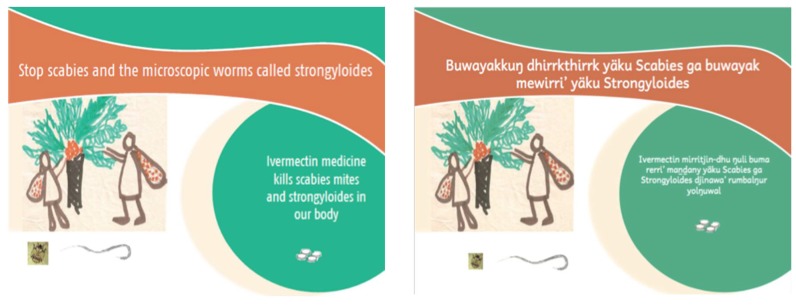
Community education resources produced by Menzies School of Health Research in English and Yolngu.

Reviews examining other barriers to strongyloidiasis control in Indigenous communities have identified several points for intervention, including the need for improved reporting protocols, increased testing of at-risk individuals, health professional engagement, and community-based monitoring and control programs [8,18,42]. Collaborative community-based initiatives incorporating mass deworming, infrastructure improvements, and culturally safe health education (Figure 6) have demonstrated significant reductions in *Strongyloides* seroprevalences [16,18,42]. One Western Australian study saw the seropositivity in 259 Indigenous adults fall from 35.3% to 5.8% in three years using these strategies [43]. A study in Arnhem Land in the Northern Territory saw seropositivity fall from 21% at baseline to 2% after 18 months of annual mass drug administration [16].

Evidence is emerging that dogs may act as hosts for human strongyloidiasis in some settings [44,45]. Animal services in remote communities are often limited, leading to animal over-population in some areas [46]. Community-based interventions may need to consider incorporating animal management into programs to address this potential reservoir [44].

Within community-based initiatives, further research is needed to inform practices relating to the testing and treatment of Indigenous children. Universal testing of Indigenous people living in endemic areas has been recommended [18,42]. The logistical challenges of implementing universal paediatric testing are substantial in our context. Paediatric blood collection is time-consuming and distressing for patients. Opportunistic blood collection is possible but carries additional costs to health services. Results are rarely available prior to discharge and locating patients for follow-up dosing and serology testing in remote communities is often difficult. Blood spot serology testing is under development and may make this investigation substantially more acceptable to parents and facilitate testing in nurse-led remote clinics where staff may have limited capacity to do paediatric venepuncture [28].

The safety and tolerability of ivermectin in paediatric patients also requires further investigation. Ivermectin is the mainstay of treatment for strongyloidiasis in adults and older children and has been used in this setting for almost 30 years [47]. The use of ivermectin in children under 15 kilograms or five years of age remains problematic due to a lack of safety data [48], although many health services (including our own) routinely use ivermectin in children under five years old and between ten and fifteen kilograms in weight, at the discretion of the treating specialist.

## 6. Conclusions

Almost 1 in 6 paediatric patients tested for strongyloidiasis at our health service were found to be seropositive. Remote communities experience an intersection of risk factors that predispose them to a disproportionate burden of disease from *S. stercoralis*. These include poorer sanitation infrastructure, inadequate and overcrowded housing, limited access to health services, very limited access to animal control services, high HTLV-1 prevalence and rates of other chronic comorbidities, and minimal disease surveillance [2,8,17,18,42].

These reflect the global experience of strongyloidiasis as a disease that predominantly affects and exploits the poorly resourced. Management of strongyloidiasis remains inextricably linked to improving the social determinants of health experienced by these communities and controlling environmental reservoirs to reduce the risk of reinfection [17].

Continued advocacy for improvements in basic infrastructure, health service resources and awareness, proactive disease monitoring, and access to effective treatment remains fundamental to the control of strongyloidiasis and other neglected diseases in the most vulnerable communities both within Australia and overseas [3,18].

## Figures and Tables

**Table 1:** Summary of original research describing *S. stercoralis* epidemiology in endemic areas in Australia.

Author	Location	Sample Size and Demographics	Years Studied	Diagnostic Test	Key Findings
Frith et al., 1974 [26]	NSW: Central Coast	Not stated	1966–1967	Stool examination	4.7% positive on stool microscopy
Jones, 1980 [26]	WA: 20 remote communities	1683 adults and children	1973–1978	Stool microscopy with formol-ether concentration	2% positive on faecal microscopy Highest infection rate in 15–19 year old age group
Prociv and Luke, 1993 [27]	QLD: 122 remote communities	Children <15 years providing 32,145 faecal samples for diagnosis and disease surveillance	1972–1991	Stool microscopy with formol-ether concentration	Overall infection prevalence of 1.97% positive Cases found in 52/122 communities Peak prevalence of 27.5% in one area during wet season vs average prevalence of 12% Reduction in prevalence from 26.2% to 7% with thiabendazole treatment of infected children
Meloni et al., 1993 [12]	WA: Kimberly region	247 adults and children in five communities	1987–1991	Stool examination	0.25% positive on microscopy 0.3% in children aged 0 to 13
Gunzburg et al., 1992 [32]	WA: Kimberly region	104 Indigenous children under 5 years old	Not stated	Stool concentration and microscopy	1.2% of samples from children with diarrhoea and 2.1% of samples from well children positive
Fisher et al., 1993 [13]	NT: Darwin	~2000 stool samples from adult and paediatric patients	1991–1992	Stool examination	68 cases of *S. stercoralis* identified 54% of diagnoses were in children under 5 years Eosinophilia noted in 57% of cases
Yiannakou et al., 1992 [33]	QLD: Townsville	14 adult and paediatric cases from 5 year audit	Not stated	Stool examination	9 Indigenous cases, 2 refugees from Vietnam, 1 returned veteran and 2 non-Indigenous patients with no significant travel history
Flannery and White, 1993 [30]	NT: Arnhem Land	29 participants	Not stated	Single stool microscopy; Serology	41% positive on faecal microscopy 59.6% positive by serological diagnosis
Shield et al., 2015 [15]	NT: Arnhem Land	314 participants including 129 children; 39 underwent serology	1994–1996	Stool microscopy; Serology	19% positive on microscopy 28% seropositive and 18% equivocal
Aland et al., 1996 [11]	NT: Arnhem Land	300 participants	Not stated	Single stool microscopy	15% positive on faecal microscopy
Page et al., 2006 [34]	NT: Arnhem Land	508 adult and adolescent participants	1996–2002	Serology	35% positive by serological diagnosis at baseline 78% seroreversion rate of cases with treatment
Kukuruzovic et al., 2002 [14]	NT: Darwin	291 children admitted with diarrhoea and 84 controls	1998–2000	Stool examination	7.2% of stool samples had *S. stercoralis* detected 87 children with wasting were 6.5 times (95% CI 1.6 to 26.7) more likely to have *S. stercoralis* Hypokalaemia significantly associated with *S. stercoralis* infection
Einsiedel et al., 2008 [35]	NT: Alice Springs	206 Indigenous adults admitted with blood stream infections	2001–2005	Serology	35.4% were positive by serological diagnosis
Einsiedel and Fernandez, 2008 [5]	NT: Alice Springs	18 Indigenous adults admitted with severe strongyloidiasis	2000–2006	Stool examination; Serology	7/11 patients with severe disease tested for HTLV-1 were positive
Einsiedel et al., 2014 [20]	NT: Alice Springs	1126 Indigenous adult inpatients	2000–2010	Serology	23.9% positive by serological diagnosis HTLV-1 positive patients trending towards higher seropositivity rates but not significant (*p* = 0.063)
Mayer-Coverdale et al., 2017 [9]	NT: Territory-wide	22,892 adult and paediatric stool samples provided to NT pathology services	2002–2012	Microscopy with formol-ether concentration	97.7% of cases Indigenous, overall 1.7% positive 42.2% of diagnoses in children under 5 years of age (3–6% positive) Declining rates of diagnosis over time noted
Kearns et al., 2017 [16]	NT: Arnhem Land	859 Indigenous children and adults	2010–2011	Microscopy/culture; Serology	21% seropositive at baseline with 15% equivocal Peak seropositivity in 5–14 year old cohort 89% patients had eosinophilia at baseline 11% had positive faecal microscopy/culture Seroprevalence 2% at 18 months after two mass drug administrations
Hays et al., 2015 [36]	WA: Kimberly region	259 Indigenous adults	2012–2015	Serology	35.3% positive by serological diagnosis (OD > 0.3) Reduction to 5.8% after three years of targeted treatment and follow up of seropositive patients

Abbreviations: NT: Northern Territory; QLD: Queensland; WA: Western Australia; NSW: New South Wales; OD: optic density; HTLV-1: human T-lymphotrophic virus 1.

**Table 2:** Demographic data, clinical presentation, and investigation results.

Variable	Seronegative (*n* = 156) Number (%)	Seropositive (*n* = 30) Number (%)	*p* Value
Mean Age	6 years 1 month	6 years 7 months	*p* = 0.55
Male Gender	91 (58.3%)	22 (73.3%)	*p* = 0.12
Remote	109 (69.9%)	27 (90.0%)	*p* = 0.02
Indigenous	149 (95.5%)	30 (100%)	*p* = 0.24
Mean serology	N/A	Optic density = 0.84 ± 1.54	
Stool pathogens	17 (36.2%), *n* = 47	5 (62.5%), *n* = 8	*p* = 0.16
Haemoglobin	117.63 ± 25.92 g/L	116.77 ± 27.22 g/L	*p* = 0.74
Mean corpuscular volume	76.578 ± 10.18 fL	76.66 ± 7.72 fL	*p* = 0.93
Mean eosinophil count *	0.96 × 10^9^/L ± 2.13 × 10^9^/L (Range 0.5 × 10^9^/L to 5.3 × 10^9^/L)	1.83 × 10^9^/L ± 1.32 × 10^9^/L (Range 0.6 × 10^9^/L to 4.8 × 10^9^/L)	*p* < 0.0001
Gastrointestinal symptoms	40 (25.6%)	9 (30%)	*p* = 0.62
Respiratory symptoms	42 (26.9%)	7 (23.3%)	*p* = 0.68
Blood stream infection	4 (2.6%)	0 (0%)	*p* = 0.37
Growth faltering	25 (16%)	3 (10%)	*p* = 0.4
HTLV-1 seroprevalence	0/10 (0%)	0/2 (0%)	

* Mean eosinophil count in patients tested for unexplained eosinophilia of ≥0.5 × 10^9^/L (seropositive group *n* = 29, seronegative group *n* = 142).

**Table 3:** Faecal examination results.

	Seronegative (*n* = 47/156) Number (%)	Seropositive (*n* = 8/30) Number (%)
Organism/virus identified	17 (36%)	5 (62.5%)
*Strongyloides stercoralis*	0	0
*Giardia* species	5	2
*Cryptosporidium parvum*	3	2
Blastocystis hominis *	1	0
Trichomonas hominis **	1	0
Entamoeba coli **	1	0
Entamoeba hartmanni **	1	0
*Salmonella* species	3	0
*Campylobacter jejuni*	1	0
Norovirus	4	0
Rotavirus	1	0
Adenovirus	1	2
*Hymenolepis nana*	1	0

* May cause clinically significant infection [38]. ** Not generally considered to cause clinically significant infections [39,40,41].
